# Metabolic Dysfunction-Associated Steatotic Liver Disease and Sarcopenia: Review of Literature

**DOI:** 10.3390/jcm15041661

**Published:** 2026-02-23

**Authors:** Hiroki Nishikawa, Soo Ki Kim, Sachiyo Yoshio, Akira Asai

**Affiliations:** 1 Second Department of Internal Medicine, Osaka Medical and Pharmaceutical University, Takatsuki 569-8686, Osaka, Japan; 2 Department of Gastroenterology, Kobe Asahi Hospital, Kobe 653-8501, Hyogo, Japan; 3 Department of Human Immunology and Translational Research, National Institute of Global Health and Medicine, Japan Institute for Health Security, Shinjukuku 162-0052, Tokyo, Japan

**Keywords:** MASLD, insulin resistance, sarcopenia, liver fibrosis, exercise

## Abstract

In 2023, the terminology of metabolic dysfunction-associated steatotic liver disease (MASLD) was proposed. MASLD uses metabolic abnormalities as an inclusion criterion. On the other hand, sarcopenia is defined by decrease in muscle mass and muscle strength. Skeletal muscle can be affected by insulin resistance (IR), and it is the largest site of insulin-stimulated glucose disposal. In recent years, advances in treatment have extended the life expectancy of patients with chronic liver disease (CLD). Due to the aging population, aging-related primary sarcopenia is expected to increase. On the other hand, liver fibrosis is an important treatment target associated with the onset of serious adverse events and poor prognosis in MASLD. The liver is the central organ for nutrition and metabolism, and patients with CLD may develop secondary sarcopenia due to various nutritional and metabolic disorders unrelated to aging. There is a strong correlation between sarcopenia, muscle fatty degeneration, liver fibrosis and IR in MASLD. MASLD and sarcopenia have a bidirectional relationship, forming a vicious cycle. In this review, we will summarize the relationship between MASLD and sarcopenia based on the current knowledge.

## 1. From NAFLD to MASLD

In 1986, Schaffner et al. proposed the concept of “nonalcoholic fatty liver disease (NAFLD)” as a general term for fatty liver disease, in which alcohol is not involved in the progression of the disease [1,2]. Subsequently, with the establishment of the concept of NAFLD, it became clear that lifestyle factors such as changes in diet and lack of exercise increase the risk of cirrhosis and cancer. In recent years, NAFLD and nonalcoholic steatohepatitis (NASH) have been on the rise. According to recent meta-analyses, the prevalence of NAFLD is estimated to be 29.7% across East Asia [3] and 22.3% in Japan [4], with approximately 10–20% of these cases considered to be NASH. In particular, cases with progressive liver fibrosis have a poor prognosis and are referred to as high-risk NAFLD. NAFLD not only increases the risk of cardiovascular events but also increases the risk of carcinogenesis in extrahepatic organs [5].

However, in recent years, concerns have been raised that the term “NAFLD” does not accurately reflect the pathophysiology of the disease. In 2020, the disease concept of metabolic dysfunction-associated fatty liver disease (MAFLD) was proposed, and in 2023, the disease concept of metabolic dysfunction-associated steatotic liver disease (MASLD) was proposed, taking into consideration the stigma associated with the disease in society [6]. MASLD uses metabolic abnormalities as an inclusion criterion, but subsequent reports from countries around the world have shown that NAFLD and MASLD are clinically almost equivalent [7,8], and the understanding that NAFLD-related clinical data can be applied to MASLD is becoming established. The disease concept and diagnostic criteria for MASLD are now internationally accepted. In 2024, resmetirom, a liver-directed and thyroid hormone receptor beta-selective agonist, was approved by the FDA for the treatment of NASH with liver fibrosis [9]. Resmetirom, at the time of approval, was the first and the only FDA-approved drug for NASH.

## 2. Recent Trend of MASLD and Sarcopenia

### 2.1. Epidemiology of MASLD and Sarcopenia

The number of MASLD patients worldwide has reached 1.27 billion, a fact that suggests this disease is a major global public health challenge [10]. MASLD is the most common chronic liver disease (CLD) in Western countries [11]. A survey of people with medical health checkups in Japan found that one in three people had MASLD, which is similar to our data [12]. It is also predicted that the number of patients with MASLD/metabolic dysfunction-associated steatotic hepatitis (MASH) will keep going up until 2035 [12]. On the other hand, there are concerns about an increase in cases of MASLD combined with sarcopenia, which is defined as quantitative and qualitative decline in skeletal muscle and/or decreased physical function [13]. Previously, the definition of sarcopenia included not only a decrease in muscle mass and strength but also a decline in physical function. However, with recent revisions by the Asian Working Group for Sarcopenia, sarcopenia is now defined solely by a decrease in muscle mass and strength, and it is recommended that physical function be measured as an outcome of sarcopenia [14]. A recent meta-analysis reported that the frequency of sarcopenia in MASLD patients was 23.5%, with a tendency to be more common in Asians and men [15]. Sarcopenia in MASLD increases in overall mortality rate associated with MASLD by 1.59 times [15]. In recent years, advances in the treatment for CLD have extended the life expectancy of patients with CLD. However, due to the aging population, aging-related primary sarcopenia is expected to increase [16]. The liver is the central organ for nutrition and metabolism, and patients with CLD may develop secondary sarcopenia due to various nutritional and metabolic disorders unrelated to aging [17].

### 2.2. MASLD/MASH as an Etiology of Liver Cirrhosis

Advanced liver fibrosis and cirrhosis are major determinants for the incidence of hepatocellular carcinoma (HCC) [18]. According to a recent meta-analysis, the pooled prevalence rates of advanced liver fibrosis and cirrhosis in the general population were 3.3% and 1.3% worldwide [19]. With the recent development of antiviral therapy and the increase in obesity, type 2 diabetes, and other metabolic diseases, the causes of cirrhosis are rapidly shifting from hepatitis virus-related liver disease to MASLD/MASH and alcohol-related liver disease (ALD). According to data released by the Japanese Society of Hepatology in 2023, alcohol-related cirrhosis accounted for 28.8% and MASH accounted for 12.7% of cases, representing a significant increase compared to previous survey results. The combined percentage of these two conditions (41.5%) significantly exceeded the percentage of hepatitis virus-related liver diseases (28%) [20]. Globally, MASLD-related advanced fibrosis, cirrhosis and HCC are also on the rise [18].

## 3. Interrelationship Between MASLD and Sarcopenia

The interrelationship between MASLD and sarcopenia is clinically very important. Precise understanding these relationships may be beneficial for clinicians.

### 3.1. The Role of Cytokine in the Development of Sarcopenia

MASLD/MASH is associated with elevated inflammatory cytokines, promoting decreased glucose utilization in muscles and increased protein catabolism. In MASLD/MASH, inflammatory cytokines such as tumor necrosis factor-α (TNF-α), transforming growth factor β (TGF-β), IL-1, and IL-6 are elevated [21]. These cytokines promote protein breakdown in skeletal muscle. In addition, growth hormone (GH) and insulin-like growth factor-1 (IGF-1) may be reduced in MASLD/MASH. GH is pituitary-derived and it stimulates hepatic IGF-1 production. Since GH and IGF-1 produced in the liver has a muscle protein-preserving effect, this may be one cause of the onset and/or progression of sarcopenia [21]. In MASLD/MASH, decreased adiponectin and hyperleptinemia due to leptin resistance are observed. Adiponectin is known to improve mitochondrial function and IR in skeletal muscle, and leptin is also known to stimulate fat burning in skeletal muscle, suggesting a possible link to the onset of sarcopenia [22]. On the other hand, Th2 cytokine IL-13 (i.e., anti-inflammatory cytokine) is induced by muscle exercise and is associated with the maintenance of muscle homeostasis [23]. Knudsen et al. reported that exercise-mediated mitochondrial biogenesis, endurance in running, and beneficial glucose effects were lost in IL-13 knockout mice, while enhanced IL-13 signaling in muscle was sufficient to improve running distance, glucose tolerance, and mitochondrial activity similar to the effects of exercise training [23]. Furthermore, IL-13 has been reported to play a crucial role in the recovery of muscle weakness associated with sepsis [24].

### 3.2. Impact of Liver Fibrosis for Sarcopenia in MASLD

In Japan, the proportion of cases with progressive liver fibrosis is expected to increase due to the aging population [20]. In MASLD patients with advanced liver fibrosis or cirrhosis, sarcopenia and/or frailty have been shown to be associated with liver fibrosis [25]. In MASLD patients, protein synthesis capacity declines with the progression of liver fibrosis, and this contributes to the onset of sarcopenia through protein catabolism in skeletal muscle [17]. The incidence of sarcopenia in patients diagnosed with MASLD/MASH by liver biopsy has been reported to range from 20.8% to 43.6%, and there is a tendency for the incidence of sarcopenia to increase as liver fibrosis progresses [7]. According to a meta-analysis of 19 cross-sectional or retrospective observational studies, the risk of sarcopenia was associated with MASLD (odds ratio (OR): 1.33), MASH (OR: 2.42), and MASLD-related liver fibrosis (OR: 1.56) [26]. In our study of MASLD patients (2093 males and 1280 females), the percentage of cases with decreased skeletal muscle mass was 15.0% in patients with FIB-4 index (liver fibrosis marker) < 1.3, 32.1% in patients with 1.3 < FIB-4 index < 2.67 and 48.3% in patients with FIB-4 index > 2.67. A close correlation between FIB-4 index and sarcopenia with the development of liver fibrosis in patients with MASLD was therefore suggested [27]. It has been reported that all-cause mortality in patients with both MASLD and sarcopenia is almost twice that of those with neither sarcopenia nor MASLD, and when advanced liver fibrosis is added to patients with both MASLD and sarcopenia, all-cause mortality is more than three times higher [28].

MASH-related HCC has been on the rise in recent years, and it has been reported that approximately 80% of MASH-related HCC patients are in a state of prefrail/frailty [29]. HCC is associated with the progression of liver fibrosis. It has been shown that frailty is more related to the quality of life in patients with cirrhosis than the severity of cirrhosis [30]. Sarcopenia is a key component of physical frailty [26]. Liver fibrosis is an important treatment target associated with the onset of serious events, poor prognosis, and decreased patient-reported outcomes in MASLD/MASH and MASH-related HCC [31,32].

### 3.3. Bidirectional Relationship Between Sarcopenia and MASLD

A large-scale cohort study of 333,295 people in the UK reported that decreased skeletal muscle mass and grip strength was associated with hospitalization and death due to MASLD [33]. Furthermore, a cohort study of 3042 people in Greece showed that MASLD patients with decreased skeletal muscle mass and higher waist circumference had a significantly higher incidence of cardiovascular disease in the future [34]. Creatinine to cystatin C ratio, which is a marker for sarcopenia, has been reported to be associated with the onset of MASLD [35]. In other words, MASLD/MASH, sarcopenia, and obesity are strongly interrelated, and individuals with sarcopenia or obesity are more likely to experience progression of MASLD, leading to an increase in liver fibrosis, cirrhosis, cardiovascular disease, and other conditions, which significantly impact overall mortality [36]. In recent years, it has been reported that patients with sarcopenia have a significantly higher risk of MASLD regardless of obesity or metabolic syndrome [37], and that the presence of sarcopenia increases the risk of MASLD by more than five times [38]. Choe et al. examined the relationship between skeletal muscle mass and MASLD and reported that sarcopenia patients had a 1.78-fold higher risk of MASLD in men and a 2.39-fold higher risk in women compared to non-sarcopenia patients [39]. Wijarnpreecha et al. conducted a meta-analysis and reported that the risk of MASLD development was significantly increased in patients with sarcopenia [40]. These results suggest a bidirectional association between MASLD and sarcopenia. The mechanisms underlying the bidirectionality between the two include IR, chronic inflammation in adipose tissue, and vitamin D deficiency, etc. [41].

There is a negative spiral between MASLD and sarcopenia, in which they adversely affect each other through factors such as physical inactivity, IR, increased inflammatory cytokines, imbalanced myokine secretion, and vitamin D deficiency, leading to a decline in liver and skeletal muscle function [42,43]. In treating MASLD patients with sarcopenia, it is important to combine weight loss with exercise therapy that simultaneously improves the function of the liver and skeletal muscles, which are important metabolic organs. The improvement of MASLD through exercise therapy is thought to result from two mechanisms: exercise promotes the release of free fatty acids from adipose tissue, which is then burned as energy in muscle cells, thereby improving IR; and it reduces lipid synthesis in the liver, leading to a decrease in hepatic fat [42,43].

### 3.4. MASLD, Circadian Clock and Sarcopenia

Circadian clock can be a regulator of muscle functions including mitochondrial metabolism and biogenesis, and MASLD patients often experience disrupted sleep–wake rhythms [44]. Recent clinical evidence indicates that circadian misalignment is linked to metabolic deregulation and disease, namely obesity [45], metabolic syndrome [46], type 2 diabetes [47], MASLD [48,49], and HCC [49].

Schaeffer et al. analyzed objective data on activity and rest recorded continuously for 24 h over four weeks using a wrist-worn accelerometer (actigram) to compare sleep patterns between MASLD patients (*n* = 35) and healthy controls (*n* = 16) [50]. The MASLD group exhibited a higher number of awakenings per night (8.5 vs. 5.5, *p* = 0.0036), longer wakefulness after sleep onset (45.4 min vs. 21.3 min, *p* = 0.0004), and lower sleep efficiency (86.5% vs. 92.8%) compared to the control group [50]. It has also been recently shown that targeting the liver circadian clock with a small molecule agonist of circadian clock gene *Rev-Erb* ameliorates liver fibrosis by regulating TGF-B signaling and features of MASH [51]. Interventions such as sleep counseling to improve the sleep–wake rhythm in MASLD patients may lead to improvements in MASLD-associated liver fibrosis and concomitant sarcopenia. Resetting the circadian clock using exercise may be helpful for the improvement of sarcopenia in MASLD [52].

## 4. Importance of IR in MASLD and Sarcopenia

IR is involved in various pathologies in MASLD. In MASLD complicated by sarcopenia, IR in skeletal muscle plays a significant role.

### 4.1. MASLD and IR

Skeletal muscle is a target organ for insulin, and in patients with sarcopenia, IR increases and serum insulin levels rise (i.e., hyperinsulinemia). Excess insulin promotes fat accumulation in hepatocytes [53]. It has also been proven that risk alleles of the representative NAFLD disease susceptibility genes (*PNPLA3*, *TM6SF2*, *GCKR*, and *MBOAT7*, etc.) increase the risk of developing type 2 diabetes [54]. *PNPLA3* and *TM6SF2* also can be associated with non-obese MASLD [54]. It has been reported that improving fat accumulation in the liver reduces the risk of developing type 2 diabetes, supporting the causal relationship between hepatic steatosis and glucose metabolism abnormalities [55]. Furthermore, a cohort study of MASLD patients reported that the higher pathological fibrosis score and steatosis score, the greater risk of developing type 2 diabetes [56]. In other words, the severer MASLD is, the worse glucose tolerance becomes.

### 4.2. Gluose Uptake in Skeletal Muscle, IR and Steatosis

**Figure 1:**
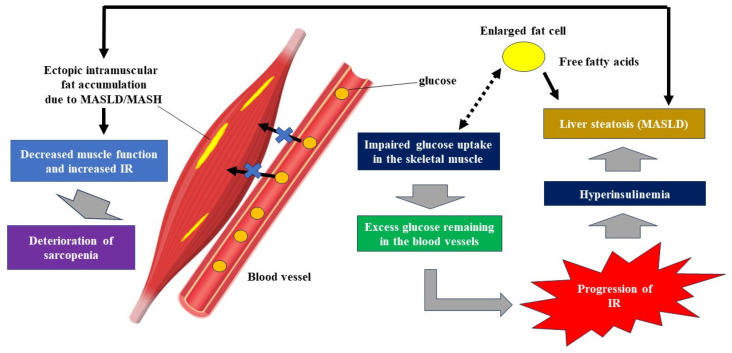
Intramuscular fat accumulation, progression of insulin resistance (IR), hepatic steatosis and sarcopenia in patients with MASLD/MASH. Under conditions of IR in skeletal muscle, glucose that is not taken up by skeletal muscle becomes a substrate for fat synthesis in liver adipose tissue, and this, combined with compensatory hyperinsulinemia, can promote hepatic steatosis. Free fatty acids are supplied to the liver from enlarged fatty tissue, which further exacerbates hepatic steatosis. Ectopic intramuscular fat accumulation due to MASLD/MASH can be associated with deterioration of sarcopenia.

Decreased functional and/or quantitative decline in skeletal muscle can be associated with insulin resistance (IR) [57]. Skeletal muscle is the largest glucose metabolism organ in the human body, processing approximately 80% of glucose [58]. A decrease in glucose uptake by muscle has been demonstrated under conditions of IR [58]. Under conditions of IR in skeletal muscle, glucose that is not taken up by skeletal muscle becomes a substrate for fat synthesis in liver adipose tissue, and this, combined with compensatory hyperinsulinemia, can promote hepatic steatosis (i.e., liver specific fatty infiltration, Figure 1). Free fatty acids are supplied to the liver from enlarged fatty tissue, which further exacerbates hepatic steatosis (Figure 1). Ectopic intramuscular fat can be accumulated due to MASLD/MASH, which leads to muscle functional decline (Figure 1). Adipose tissue also produces inflammatory proteins and cytokines such as CRP, TNF-α, IL-6, and IL-1β, creating a chronic inflammatory environment that contributes to muscle atrophy and the progression of sarcopenia [43]. On the other hand, the relationship between fat mass, skeletal muscle mass, and IR is also important. In our study, we found a significant positive correlation between Homeostasis Model Assessment of Insulin Resistance and fat mass to skeletal muscle mass ratio [59]. In other words, if both fat mass and skeletal muscle mass decrease, and muscle mass decreases more than fat mass, it will not contribute to improving IR. Effective improvement in IR can be achieved by reducing fat mass and increasing skeletal muscle mass through exercise and other means.

### 4.3. Hepatic Steatosis, IR and the Role of Hepatokines

Hepatic steatosis is associated with IR in skeletal muscle and indicators related to basal glucose production in the liver. There is a negative correlation between hepatic steatosis and insulin sensitivity in skeletal muscle. It is hypothesized that hepatokines secreted from the liver may be responsible for the tendency of hepatic steatosis to reduce insulin sensitivity in skeletal muscle (Table 1). Leukocyte cell-derived chemotaxin 2 (LECT2) is a hepatokine, and its blood concentration is positively correlated with body mass index (BMI), waist circumference, and liver fat accumulation, and its expression is suppressed by AMP-activated protein kinase (AMPK) activation. Its expression increases in response to overnutrition. LECT2 secreted from the liver induces IR in skeletal muscle through c-jun N-terminal kinase phosphorylation [60]. On the other hand, hepatic steatosis is not associated with insulin sensitivity in the liver [61]. Selenoprotein P is another hepatokine that links hepatic steatosis and IR in skeletal muscle. Selenoprotein P is suppressed by insulin, but its expression increases in response to higher glucose and free fatty acids, causing IR in the liver and skeletal muscle. Furthermore, Selenoprotein P inactivates AMPK by eliminating exercise-induced reactive oxygen species in skeletal muscle, thereby inhibiting the acquisition of aerobic exercise capacity [62,63]. Other hepatokines include FetuinA and Hepassocin, both of which are known to increase expression in hepatic steatosis and induce IR in the liver and skeletal muscle [64]. Fetuin-A acts as an endogenous ligand for TLR4 and promotes lipid-induced IR [65]. Overexpression of Hepassocin is reported to induce fat accumulation via an extracellular signal-regulated kinase 1/2 (ERK1/2)-dependent pathway [66]. Conversely, FGF-21 has been reported as a hepatokine that decreases in expression with hepatic steatosis and enhances insulin sensitivity [67]. FGF-21 promotes glucose uptake by adipocytes in an insulin-independent manner [68]. This organ-organ interaction, whereby liver with hyper-nutritional status induces IR in skeletal muscle via humoral factors, is thought to be one of the mechanisms that exacerbates glucose tolerance in MASLD patients.

### 4.4. Sarcopenia, Myokines and IR

Since Pedersen et al. discovered myokines secreted from skeletal muscle as biomarkers in 2003, exercise has come to be recognized not only as a means of disease prevention but also as a form of treatment [69]. The endocrine function of muscles is attracting attention as a mechanism by which exercise improves liver fibrosis. Cytokines and peptides secreted from muscle tissue during muscle contraction are called myokines and interact with multiple organs, including the liver [70]. Some myokines induce anti-inflammatory responses during exercise, and long-term exercise is thought to improve cardiovascular risk [69]. Furthermore, it has been clarified that myokines have a wide range of effects on the entire body, including glucose production in the liver, glucagon like peptide-1 (GLP-1) expression in the intestines, cognitive function, lipid and glucose metabolism, browning of white adipocytes, bone formation, vascular endothelial cell function, skin structure, and tumor proliferation [69]. Representative myokines include IL-6, myostatin, irisin, brain-derived neurotrophic factor (BDNF), and decorin (Table 2) [71]. IL-6 was defined as a myokine by Pedersen. IL-6 has been demonstrated that it is a myokine transiently secreted from skeletal muscle cells in response to muscle contraction [69]. IL-6 is associated with IR and chronic inflammation in skeletal muscle of MASLD patients [72]. In other words, there is exercise-induced transient IL-6 release and chronically elevated IL-6 in inflammatory states. Myostatin is a myokine belonging to the TGF-β family [73]. Myostatin is associated with skeletal muscle mass, and mice lacking myostatin have 2–3 times more skeletal muscle mass, indicating that myostatin strongly inhibits muscle protein synthesis [73]. In patients with cirrhosis, higher serum myostatin levels and sarcopenia have been reported to be associated with poor prognosis [74]. Irisin is a myokine involved in energy metabolism that promotes heat production and browns adipose tissue [75]. BDNF is a myokine that promotes fatty acid oxidation and is associated with systemic metabolism [76,77]. BDNF is also associated with liver fibrosis [78]. Decorin has been reported to have an inhibitory effect on liver carcinogenesis, tumor invasion, and metastasis [79]. It has been clarified that decorin is related to skeletal muscle mass in patients with HCC [8]. IL-6, myostatin, and irisin are involved in glucose and fatty acid metabolism in hepatocytes [80]. In recent years, development of anti-myostatin antibody drugs and other treatments for sarcopenia has been progressing [81].

### 4.5. Sarcopenia, Vitamin D and IR

Experimental animal studies have shown that vitamin D deficiency deteriorates liver inflammation and steatosis, indicating a potential protective role against MASLD [82]. Furthermore, recent studies have revealed that vitamin D deficiency is associated with sarcopenia [83]. Vitamin D is a fat-soluble vitamin that is obtained from food and also biosynthesized in the skin through exposure to ultraviolet light. Decreased serum vitamin D (25(OH)D) levels have been reported to be associated with: (1) reduced muscle strength and mass [83], (2) increased risk of falls [84], and (3) increased susceptibility to obesity [85]. Vitamin D suppresses the expression of genes associated with skeletal muscle atrophy, thereby improving IR in skeletal muscle [83].

## 5. MASLD and Sarcopenic Obesity, and Non-Obese MASLD and Sarcopenia

### 5.1. MASLD and Sarcopenic Obesity

In MASLD/MASH, obesity plays a central role in the onset and progression of the disease, while “sarcopenic obesity” which is accompanied by sarcopenia, is also attracting attention. Sarcopenia or sarcopenic obesity in MASLD/MASH has different characteristics from sarcopenia in cirrhosis with malnutrition, so it is important to optimize nutritional and exercise therapy for each condition. In a condition called “sarcopenic obesity,” where both obesity and sarcopenia are present, achieving two conflicting goals (i.e., reducing weight and maintaining muscle mass) is considered an important factor in improving prognosis [86]. It has been reported that obese MASLD/MASH patients are prone to sarcopenia despite higher BMI (the incidence of sarcopenia is 20.8–43.6%, and the incidence increases with the progression of liver fibrosis [38,87]), and it is speculated that IR arises due to the atrophy of skeletal muscle, an insulin target organ, which is deeply involved in the pathophysiology [38,87]. IR and hyperinsulinemia promote gluconeogenesis in hepatocytes and inhibit glycogen production. Furthermore, the uptake of free fatty acids into the liver increases, promoting fat deposition in hepatocytes through enhanced fat synthesis and inhibition of β-oxidation [88]. On the other hand, in skeletal muscle, gluconeogenesis promotes muscle protein breakdown, and in a higher insulin state, mechanistic target of rapamycin in muscle is suppressed, resulting in the promotion of autophagy and the onset or worsening of sarcopenia [88].

### 5.2. Non-Obese MASLD and Sarcopenia

The number of non-obese MASLD patients has also been increasing in recent years, and it is urgent to elucidate the pathogenesis of non-obese MASLD [89,90]. Lean MASLD is MASLD without obesity. It is estimated that approximately 20% of MASLD cases are lean MASLD, but there are many unknowns regarding its pathology and treatment [7]. It has been reported that an increase in the energy ratio of carbohydrates and a decrease in physical activity are predictors of lean MASLD, regardless of BMI or total energy intake [91]. A meta-analysis comparing the prognosis of non-obese and obese MASLD patients found that there was no significant difference in all-cause mortality (OR: 1.09, 95% confidence interval [CI]: 0.66–1.90), cardiovascular mortality (OR: 1.12, 95% CI: 0.66–1.90), liver-related events (decompensated cirrhosis or hepatocellular carcinoma) (OR: 0.81, 95% CI: 0.50–1.30), and all were found to have equivalent risks regardless of obesity status. However, the OR for liver-related death was 1.88 (95% CI: 1.02–3.45), indicating that non-obese MASLD is associated with a higher risk of liver-related death than obese MASLD [92]. IR plays a central role as a risk factor for MASLD, and multiple factors such as dietary intake, lack of exercise, visceral fat, muscle, genetic factors, and changes in the intestinal microbiota are complexly interrelated [93]. Furthermore, adiponectin, which functions in muscle cells to enhance the metabolism of glucose and fats, is reduced to the same extent in lean MASLD and non-lean MASLD [94], indicating that excessive accumulation of visceral fat is a contributing factor to the pathogenesis of lean MASLD. As described earlier, sarcopenia, muscle fatty degeneration, and IR are strongly related. Sarcopenia causes poor serum glucose control by promoting IR and reducing the amount of muscle required for glucose uptake. A decrease in energy consumption due to reduced physical activity is also a factor that worsens IR. Increased IR inhibits muscle regeneration and causes chronic inflammation, and TNF-α and IL-6 inhibit muscle protein synthesis and promote muscle protein degradation. Furthermore, an increase in myostatin also increases liver fat [95]. To date, it has been reported that sarcopenia is associated with MASLD, MASH, and liver fibrosis independently of BMI and metabolic abnormalities [26,43,96]. Lean MASLD has been reported to have less muscle mass than obese MASLD [97], which is consistent with our findings [98].

Shoda’s research group identified visceral fat area, HbA1c, myostatin, and leptin as factors influencing liver fat accumulation in non-obese MASLD. Leptin is a protein produced in visceral adipose tissue that is involved in appetite suppression and energy metabolism regulation [99]. Furthermore, their research revealed that non-obese MASLD patients often have visceral fat obesity, and that many cases with advanced liver fibrosis were observed in non-obese women with MASLD [99]. In our cross-sectional study of MAFLD patients (2014 men and 949 women), the proportion of cases with decreased skeletal muscle mass was strongly correlated with BMI. In men, the proportion of cases with decreased skeletal muscle mass was 100% in the BMI < 20 kg/m^2^ group, 31.7% in the 20 kg/m^2^ < BMI < 25 kg/m^2^ group, and 0.4% in the BMI > 25 kg/m^2^ group (*p* < 0.0001). In women, the proportion of cases with decreased skeletal muscle mass was 96.7% in the BMI < 20 kg/m^2^ group, 21.1% in the 20 kg/m^2^ < BMI < 25 kg/m^2^ group, and 0% in the BMI > 25 kg/m^2^ group (*p* < 0.0001). In multivariate analysis, BMI was an independent factor correlated with the FF index (fat-free mass/(height)^2^ (kg/m^2^) in both men and women [98]. In cases with non-obese MASLD with BMI < 20 kg/m^2^, particular attention should be paid to the coexistence of sarcopenia. In addition to muscle mass, attention should also be paid to the deterioration of muscle quality associated with myosteatosis [100,101]. It has been reported that impaired fat breakdown in muscles may lead to intramuscular fat accumulation [102]. Intramuscular fat accumulation in patients with MASLD/MASH can be associated with progression of IR (Figure 1) [102].

## 6. Met-ALD, ALD and Sarcopenia

### 6.1. Alcohol Intake, Disease Progression and Mechanisms for Sarcopenia

Drinking alcohol increases the risk of various diseases. Hypertension and lipid metabolism abnormalities are dependent on the amount of alcohol consumed, while liver cirrhosis and cardiovascular disease are associated with a sharp increase in the risk of disease development when alcohol consumption exceeds a certain threshold [103]. The risk of progression to decompensated cirrhosis over 5 years has been reported to be <1% for MASLD with F1 (i.e., mild liver fibrosis), 1–5% for metabolic dysfunction and alcohol-associated liver disease (Met-ALD; moderate drinker) with F1, 2–15% for ALD (heavy drinker) with F1, 5–15% for MASLD with F3 (i.e., advanced liver fibrosis) or severer, 10–30% for Met-ALD with F3 or severer, and 15–50% for ALD with F3 or severer [104]. Alcohol intake and life expectancy are closely correlated above a certain amount of alcohol intake [105]. Heavy drinking has negative effects on skeletal muscle due to decreased food intake other than alcohol (i.e., starvation), direct damage to skeletal muscle by acetaldehyde, decreased ammonia clearance, increased intestinal permeability, gonadal hormone abnormalities, and GH abnormalities [106]. Alcohol consumes a large amount of energy during its metabolism in the liver. As a result, the energy supplied to the muscles becomes insufficient. Alcohol consumption increases susceptibility to dehydration and electrolyte imbalances, adversely affecting muscle cell function. Long-term alcohol intake leads to elevated inflammatory cytokines and muscle cell damage [105]. Particularly, ethanol suppresses ATP production by inhibiting mitochondrial function in muscle cells. Furthermore, ethanol and acetaldehyde reduce skeletal muscle mass by both inhibiting muscle protein synthesis and promoting degradation. Therefore, abstinence or moderation in alcohol consumption is an effective measure for improving sarcopenia [107].

### 6.2. Clinical Implication for Alcohol Intake on Sarcopenia

Heavy drinking in middle age and early old age was reported to potentially increase the risk of sarcopenia and frailty. It has been reported that skeletal muscle mass peaked at moderate amounts of alcohol intake, but as intake increased further, skeletal muscle mass continued to decline [105]. In a previous study of our SLD patients (2435 men and 1207 women), we classified them into four groups according to their alcohol consumption: non-drinkers (i.e., MASLD), light drinkers (i.e., MASLD), moderate drinkers (i.e., Met-ALD), and heavy drinkers (i.e., ALD), and examined their relationship to skeletal muscle mass [108]. The average FF index in non-drinkers, light drinkers, Met-ALD, and ALD in men were 19.01, 19.29, 18.50, and 18.55 kg/m^2^ (overall *p* < 0.0001), and those in women were 16.03, 15.96, 15.62, and 15.07 kg/m^2^ (overall *p* < 0.0001). The results showed that skeletal muscle mass was significantly lower in moderate and heavy drinkers regardless of gender, suggesting a link between alcohol consumption and the development of sarcopenia [108]. From the perspective of skeletal muscle, light alcohol consumption appears to have few adverse effects.

## 7. Sarcopenia and Antidiabetic Drugs in MASLD

It may be necessary to mention drug-induced sarcopenia. In recent years, significant progress has been made in diabetes treatment through antidiabetic drugs. Sodium glucose cotransporter 2 (SGLT2) inhibitors are diabetes medications that lower serum glucose levels independently of insulin by promoting glucose excretion in urine [109]. Recently, their multifaceted effects on the cardiovascular system, kidneys, and liver have become apparent, and chronic heart failure and chronic kidney disease have been added to its indications. In addition, it has been shown to have a beneficial effect on patients with liver dysfunction and fatty liver, and the 2020 NAFLD/NASH Treatment Guidelines now recommend SGLT2 inhibitors for NAFLD/NASH patients with type 2 diabetes [7]. SGLT2 inhibitors may mobilize amino acids from skeletal muscle by promoting gluconeogenesis in the liver and may also cause weight loss due to urinary glucose excretion. Therefore, caution is required regarding sarcopenia-related complications in the elderly [63]. In animal experiments, there have been reports that SGLT2 inhibitors suppress skeletal muscle atrophy; however, clinical trials have not reached a consensus on changes in muscle mass and muscle strength, and further investigation is needed [110]. A recent meta-analysis of patients with type 2 diabetes showed that SGLT2 inhibitors suppressed weight, BMI, and waist circumference reduction, but significantly reduced muscle mass [109]. On the other hand, in recent years, the GLP-1 receptor agonist, semaglutide (once-weekly subcutaneous semaglutide at a dose of 2.4 mg for 240 weeks), has been reported to improve fatty liver and liver fibrosis in patients with MASLD in the phase 3 trial [111]. This study reported an average weight loss of 10.5% in the semaglutide group [111]. Severe weight loss can be associated with sarcopenia [112] and there are concerns about the onset or progression of sarcopenia, especially in Japan, where there are many elderly patients with MASLD. On the other hand, metformin, frequently used for type 2 diabetes, has been reported to exert a protective effect against sarcopenia and frailty in numerous studies [113].

## 8. Effect of Chronic Inflammation in the Spleen for Sarcopenia in MASLD

The spleen is one of the organs crucial for regulating cytokine production throughout the body. The spleen is a key immunoregulatory organ associated with sarcopenia via chronic inflammation. Inflammatory cytokines such as IL-6 and TNF-α originating from the spleen directly inhibit muscle regeneration and muscle protein synthesis, thereby promoting muscle wasting. Hypersplenism further exacerbates this vicious cycle, making spleen function a critical factor in maintaining muscle mass [114]. Anemia associated with hypersplenism promotes sarcopenia [115], and leukopenia associated with hypersplenism may lead to sarcopenia due to increased susceptibility to infection resulting from impaired immunity [116]. In MASLD, metabolic stressors such as IR and obesity induce a state of low-grade chronic inflammation throughout the body [117]. In MASLD, spleen volume correlates with liver volume and the degree of liver fibrosis [118]. In other words, in MASLD with advanced liver fibrosis, spleen function becomes hyperactive, leading to increased activity of spleen-derived inflammatory signals, which enhances the risk of developing sarcopenia (i.e., liver-spleen-muscle crosstalk).

## 9. Nutritional Therapy and Exercise in MASLD with Sarcopenia

### 9.1. Fundamental Nutritional Approach for MASLD and Sarcopenia

Decreased physical activity is one of the main factors in the onset of MASLD [119]. When considering nutritional dietary therapy for MASLD/MASH, it is necessary to evaluate obesity, sarcopenia, and liver reserve capacity, and to consider the appropriate calorie intake and nutritional composition. For patients with obesity-related MASLD/MASH, the primary treatment is a low-calorie diet that restricts carbohydrates or lipids. Weight loss is known to improve the condition of MASLD/MASH. Specifically, a 5% reduction in weight has been shown to improve quality of life, a 7% reduction to improve histological features such as hepatic steatosis, inflammatory cell infiltration, and ballooning degeneration, and a 10% reduction to improve liver fibrosis [7]. However, for MASLD/MASH patients with sarcopenic obesity, it is necessary to carefully consider the approach to calorie restriction, taking into account liver reserve capacity and skeletal muscle mass. It is generally known that weight loss achieved solely through energy restriction further induces a decrease in skeletal muscle mass. Therefore, for sarcopenic obesity, it is recommended to combine energy restriction with resistance exercise and adequate protein intake. In type 2 diabetes patients who are prone to sarcopenic obesity, there is no relationship between protein intake and mortality risk in those under 75 years of age, but it has been reported that mortality risk increases in those over 75 years of age with protein intake < 1.15 g/kg/day [120]. In addition, it has been reported that individuals with a BMI > 28 kg/m^2^ are at high risk of decline in activity of daily life [121], and interventions aimed at maintaining or normalizing weight in frailty and/or sarcopenic individuals with energy intake and weight problems are important.

### 9.2. Nutritional Approach in Liver Cirrhosis

It has been reported that weight loss improves outcomes in obese patients with compensated cirrhosis [122]. On the other hand, patients with decompensated cirrhosis have impaired energy metabolism, increased protein catabolism, and decreased protein synthesis. Therefore, extreme calorie restriction and protein restriction may promote protein and energy malnutrition and sarcopenia. In fact, weight loss surgery is an effective means for achieving weight loss by restricting calorie intake, but it has been reported that in patients with decompensated cirrhosis, it induces sarcopenia and malnutrition, increasing the risk of poor prognosis before liver transplantation [123]. From this, it is desirable that in MASLD/MASH patients with obesity, in order to achieve both weight loss and maintenance of muscle mass, calorie restriction focused on weight loss should be performed in patients with compensated cirrhosis. In patients with decompensated cirrhosis, extreme calorie restriction should be avoided, an appropriate calorie intake should be maintained, and exercise therapy should be combined while ensuring protein intake. On the other hand, The Japanese Society for Clinical Nutrition and Japanese Association on Sarcopenia and Frailty recently issued the “Nutrition Management Guidelines for Sarcopenia and Frailty 2025.” Among these, branched chain amino acid preparations, carnitine preparations, zinc preparations, vitamin D, and β-hydroxy β-methylbutyrate administration are recommended for the prevention and treatment of sarcopenia and/or frailty associated with cirrhosis.

### 9.3. Exercise and MASLD

Exercise therapy is a fundamental treatment for MASLD/MASH. Exercise therapy is strongly recommended in the Japan 2020 NAFLD/NASH Clinical Practice Guidelines [7]. Regular exercise reduces intrahepatic fat content, suppresses the onset of liver fibrosis and HCC, and prolongs survival in MASLD patients [124]. As physical activity, the American Gastroenterological Association recommends 150–300 min/week (20–40 min/day) of exercise at 3.6 metabolic equivalents (METs) or 75–150 min/week (10–20 min/day) of exercise at 6 METs or higher [125]. A systematic review of physical activity aimed at reducing visceral fat found that aerobic exercise intervention (i.e., light jogging, etc.) significantly reduced visceral fat and that at least 10 METs × hours/week of aerobic exercise was necessary [126]. Both aerobic exercise and resistance training are effective for MASLD. From the perspective of exercise characteristics, it seems desirable to engage in a combination of exercises not only for the effects on liver disease but also for health promotion [127]. Many patients with liver disease have poor exercise tolerance and lack exercise habits, so a variety of exercise programs are needed to enable as many patients as possible to engage in exercise. In addition, as mentioned above, Japanese patients with liver disease are becoming significantly older, and thus it is necessary to pay close attention to falls during exercise therapy. When performing exercise therapy, it goes without saying that it is most important to ensure that the benefits of exercise outweigh the risks. Overseas, more than 90% of liver specialists, gastroenterology specialists, and diabetes specialists recognize the usefulness of exercise therapy and recommend exercise to their patients, but it has been reported that only about 20% of patients actually receive exercise guidance [128]. We should actively promote awareness of the usefulness of exercise therapy.

### 9.4. Natural Product, Herbal Agents and Sarcopenia in MASLD

There is evidence supporting the significant beneficial effects of several plant-de rived natural products on sarcopenia, including catechins [129], resveratrol [130], soy protein [131], curcumin [132], and ginseng [133]. Herbal agents have been demonstrated to yield beneficial effects on various indicators used for the diagnosis of sarcopenia, including physical performance, handgrip strength, weight-lifting capacity, time/distance feeling tired, skeletal muscle mass and resistance to muscle fatigue, etc. [134]. Several studies have reported that oxidative stress, inflammation, and activity of daily life all improved by the regular ingestion of herbal bioactive compounds [134]. Similar effects may be expected in MASLD patients with sarcopenia, although clinical evidence is limited.

## 10. Final Remarks

**Figure 2:**
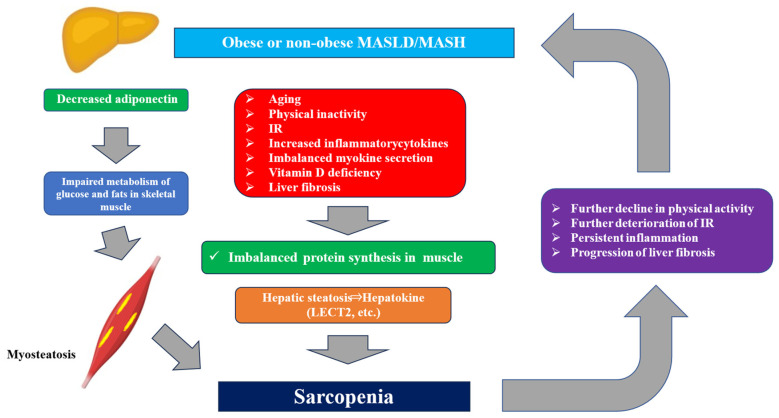
The relationship and mechanism between MASLD and sarcopenia. Various factors contribute to the development of sarcopenia in MASLD/MASH patients. MASLD/MASH and sarcopenia have a bidirectional relationship, forming a vicious cycle. IR; insulin resistance, LECT2; Leukocyte cell-derived chemotaxin 2.

Based on previous reports, we summarized the relationship between MASLD and sarcopenia. MASLD and sarcopenia are interrelated, and various molecular mechanisms underlie this relationship, forming a vicious cycle. There is a strong correlation between sarcopenia, muscle fatty degeneration, liver fibrosis and IR. Whether it is obese MASLD or non-obese MASLD, it is important to note that sarcopenia is frequently associated with both types and is a poor prognostic factor. Appropriate nutritional and exercise interventions tailored to the condition can improve prognosis. Finally, the relationship and mechanism between MASLD and sarcopenia are shown in Figure 2.

## Figures and Tables

**Table 1:** Hepatokines that modulate insulin resistance in MASLD.

Hepatokine	IR in Skeletal Muscle	Main Characteristics	Main Target	Refs.
LECT2	Deterioration	c-jun N-terminal kinase phosphorylation	obesity-related IR	[60]
Selenoprotein P	Deterioration	Inactivation of AMP kinase	obesity-related IR	[62,63]
FetuinA	Deterioration	Endogenous ligands of TLR4	obesity-related IR	[64,65]
Hepassocin	Deterioration	ERK1/2-dependent pathway	obesity-related IR	[64,66]
FGF-21	Improvement	Insulin-independent action	obesity-related IR	[67,68]

IR; insulin resistance, LECT2; Leukocyte cell-derived chemotaxin 2, ERK1/2; extracellular signal-regulated kinase 1/2.

**Table 2:** Summary of myokines.

Myokine	Clinical Implication	Primary Metabolic Endpoint	Refs.
IL-6	Chronic elevation due to inflammation	Induction of IR in skeletal muscle	[72]
Myostatin	Exercise-induced decrease	Inhibition of muscle protein synthesis	[73]
Irisin	Promotion in heat production	Improvement in energy metabolism	[75]
BDNF	Promotion in fatty acid oxidation	Improvement in systemic metabolism	[76,77]
Decorin	Exercise-induced increase	Promotion of muscle protein synthesis	[8]

BDNF; brain-derived neurotrophic factor, IR; insulin resistance.

## Data Availability

No new data were created or analyzed in this study. Data sharing is not applicable to this article.

## Abbreviations

NAFLD; nonalcoholic fatty liver disease, NASH; nonalcoholic steatohepatitis, MAFLD; metabolic dysfunction-associated fatty liver disease, MASLD; metabolic dysfunction-associated steatotic liver disease, CLD; chronic liver disease, MASH; metabolic dysfunction-associated steatohepatitis, IR; insulin resistance, HCC; hepatocellular carcinoma, ALD; alcohol-associated liver disease, TNF-α; tumor necrosis factor-α, GH; growth hormone, IGF-1; insulin-like growth factor-1, TGF-β; transforming growth factor β, OR; odds ratio, LECT2; leukocyte cell-derived chemotaxin 2, BMI; body mass index, AMPK; AMP-activated protein kinase, SGLT2; sodium glucose cotransporter 2, GLP-1; glucagon like peptide-1, BDNF; brain-derived neurotrophic factor, CI; confidence interval, Met-ALD; metabolic dysfunction and alcohol-associated liver disease. METs; metabolic equivalents
